# Outcomes and Control Rates for I-125 Plaque Brachytherapy for Uveal Melanoma: A Community-Based Institutional Experience

**DOI:** 10.1155/2014/950975

**Published:** 2014-03-09

**Authors:** Aaron Wagner, Andy Chen, Taylor Cook, David Faber, Kirk Winward, William Sause

**Affiliations:** ^1^Department of Radiation Oncology, Huntsman Cancer Institute, University of Utah, Salt Lake City, UT 84112, USA; ^2^Department of Radiation Oncology, Intermountain Medical Center, 5131 Cottonwood Street, Murray, UT 84107, USA; ^3^Rocky Mountain Retina Associates, Murray, UT 84107, USA; ^4^Retina Associates of Utah, Murray, UT 84107, USA

## Abstract

*Purpose*. To evaluate our community-based institutional experience with plaque brachytherapy for uveal melanomas with a focus on local control rates, factors impacting disease progression, and dosimetric parameters impacting treatment toxicity. *Methods and Materials*. Our institution was retrospectively reviewed from 1996 to 2011; all patients who underwent plaque brachytherapy for uveal melanoma were included. Follow-up data were collected regarding local control, distant metastases, and side effects from treatment. Analysis was performed on factors impacting treatment outcomes and treatment toxicity. *Results*. A total of 107 patients underwent plaque brachytherapy, of which 88 had follow-up data available. Local control at 10 years was 94%. Freedom from progression (FFP) and overall survival at 10 years were 83% and 79%, respectively. On univariate analysis, there were no tumor or dosimetric treatment characteristics that were found to have a prognostic impact on FFP. Brachytherapy treatment was well tolerated, with clinically useful vision (>20/200) maintained in 64% of patients. Statistically significant dosimetric relationships were established with cataract, glaucoma, and retinopathy development (greatest *P* = 0.05). *Conclusions*. Treatment with plaque brachytherapy demonstrates excellent outcomes in a community-based setting. It is well tolerated and should remain a standard of care for COMS medium sized tumors.

## 1. Introduction

Uveal melanoma is an uncommon cancer, with age-adjusted incidence rates of 4.3 new cases per million [1]. Mortality however is not rare, with metastases present in up to 20–39% of patients at 20 years, and tumor related death ranging from 17–20% at 20 years [2]. There have been multiple investigations into appropriate treatment options, and current accepted standards range from observation to enucleation, all dependent on the size and characteristics of the tumor [3–5].

Brachytherapy is frequently utilized for medium sized tumors (apical height 3–10 mm and basal diameter 5–16 mm) and has been shown to be equivalent to enucleation for tumors in this category [3]. Treatment delivery is nevertheless quite complex, and it has been recommended to only undertake this treatment approach at medical centers with the appropriate expertise [6]. Accordingly, recommendations have been made by the American Brachytherapy Society regarding appropriate treatment delivery and planning [6].

While the efficacy of plaque brachytherapy has been well established in large institutional practices that are well versed in its implementation [7–9], smaller institutional and community-based results are not as readily available. In addition, while the long term outcomes from plaque brachytherapy have been well established [3, 8–10], the literature does not have very well-documented local control rates at long term follow-up [11] to characterize the time at which failures occur. Lastly, comparisons of dosimetric and treatment planning parameters to both treatment toxicity as well as outcomes are not as well recognized, being limited to a select few studies [8, 12–15].

The purpose of this study was to evaluate our institutional community-based practice of brachytherapy for uveal melanomas. This included looking at survival rates, local control and metastatic rates, and the requirements for salvage treatment. In addition, further investigations were performed to evaluate the factors which predicted progression, as well as dosimetric and tumor factors which predicted ocular complications.

## 2. Methods and Materials

Plaque brachytherapy has been utilized for uveal melanomas at our institution since 1996 and has been performed in a community-based setting. Institutional review board and ethics committee approval was obtained, and patients treated between 1996 and 2011 were retrospectively reviewed, and all patients who underwent plaque brachytherapy for uveal melanoma were included. Data was collected regarding patient demographic data, tumor characteristics, and treatment characteristics. Patients were followed with the treating ophthalmologist, and follow-up data were collected regarding local tumor control, salvage treatment requirements, treatment side effects, occurrence of metastases, and survival status. Tumor characteristics were classified according to the COMS classification, with medium sized tumors having a height of 2.5–10 mm and diameter of 5–16 mm, large tumors a height of >10 mm or diameter >16 mm, and small tumors heights of <2.5 mm and not meeting requirements for medium or large size tumors. AJCC stage was assigned per the AJCC 7th edition staging.

Patients were diagnosed by fundoscopic exam, fluorescein angiography, and B-scan ultrasound examination. Tumor characteristics were recorded including tumor dimensions, location, and distance from intraocular structures.

Plaque implants were performed in accordance with American Brachytherapy Society recommendations [6]. Patients were evaluated by one of three ophthalmologists trained in plaque brachytherapy and our institutional radiation oncology department. Tumors were localized by indirect ophthalmoscopy and occasionally fundoscopic photography in clinic and transillumination at the time of surgery. Plaques of the COMS type were chosen with an additional tumor base margin of at least 2 mm. Treatment was planned in accordance with the American Association of Physicists in Medicine (AAPM) Updated Task Group Number 43 Report. Dose calculations were performed per the COMS protocol using the Bebig Plaque Simulator software. The sources were treated as point sources without corrections for anisotropy, silastic carrier attenuation, scatter from the gold plaque, or L-shell fluorescence. Dose points were carried at the tumor apex, the center of the optic disk, the center of the lens, the scleral surface, and the macula. Iodine-125 (I-125) was utilized in all cases, with a planned dose of 85 Gy to the tumor apex, or to a 5 mm depth when tumors were less than 5 mm in height.

Surgery was performed by the referring ophthalmologist with a radiation oncologist and physicist present. Plaque position was verified either with a dummy plaque or indirect ophthalmoscopy with scleral depression. Muscle transposition was performed if necessitated by tumor position. Plaques were sutured in place and left in place for a time interval as dictated by source strength and desired dose, before being removed.

Planned follow-up was with the treating ophthalmologist, with scheduled appointments planned for 3 to 6 months during the first year, followed by 6 months to annually thereafter. Patients underwent repeat fundoscopic examination, as well as yearly B-scan ultrasound evaluation. Yearly hepatic enzyme evaluation and liver ultrasound as indicated were also performed.

Statistical analysis was performed using StatsDirect statistical software (version 2.7.8, Stats Direct Ltd., Altrincham, UK). Survival was calculated from time of the implant. Kaplan-Meier curves were generated for both overall survival and freedom from progression (OS and FFP, resp.), as well as independently for local control (LC) rates and freedom from distant metastases. FFP was defined as simultaneous local control and the absence of distant metastases. Local control was evaluated by the treating ophthalmologist with repeat fundoscopic examination, and control was defined as no visible tumor growth. If any further salvage treatment was performed, such as laser photocoagulation, this was deemed a failure even in the absence of tumor growth. Univariate analysis was performed by log rank analysis to identify factors impacting FFP. Statistical significance of factors affecting side effect rates was performed with an unpaired *t*-test for continuous variables and Pearson *χ* ^2^ test for categorical variables. Statistical significance was defined at *P* = 0.05.

## 3. Results

Between April of 1996 and November of 2011, 107 patients underwent plaque brachytherapy for uveal melanoma for which dosimetric data were available. Of these, 88 patients had data regarding treatment follow-up. Patient characteristics are displayed in Table 1. For all patients, 85% of tumors were in the COMS medium sized category, with the majority of the others in the small category. The mean largest diameter was 10.67 mm (5.70–18.30 mm), the mean height was 4.49 mm (1.60–10.17 mm). The mean distance to the macula was 6.07 mm (0–15.73 mm), and the mean distance to the optic disk was 5.93 mm (0.01–17.37 mm).

I^125^ was utilized for all seed implants, and the most common plaque utilized was the COMS 14 size. The average number of seeds was 14 (range 5–24). The average treatment time was 102.11 hours, with an average dose rate of 0.92 Gy/hr to the tumor apex. The average seed activity was 3.2 mCi. The actual delivered treatment dosimetric characteristics are displayed in Table 2.

**Figure 1:**
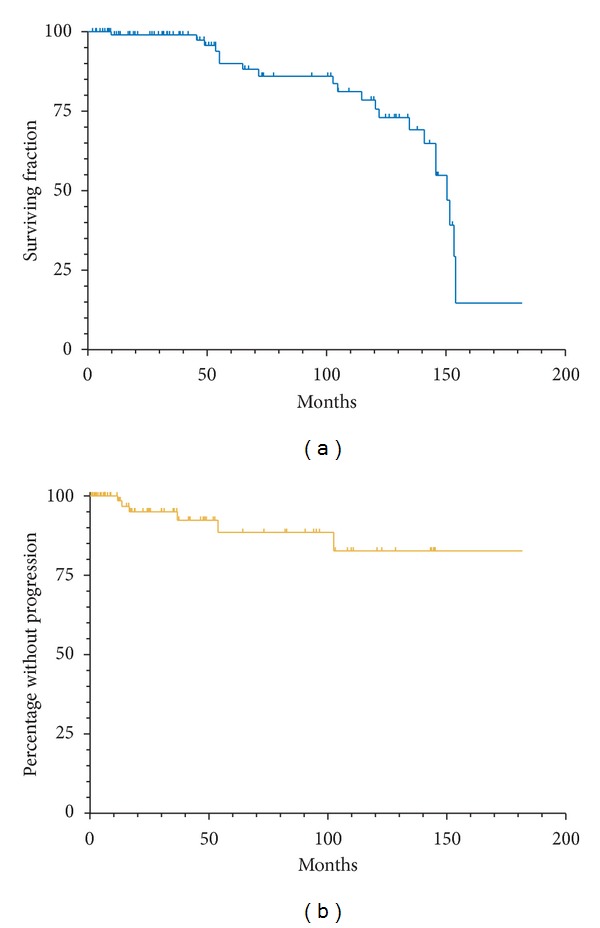
(a) Overall survival of patient population. (b) Freedom from progression from either local disease or distant metastases.

**Figure 2:**
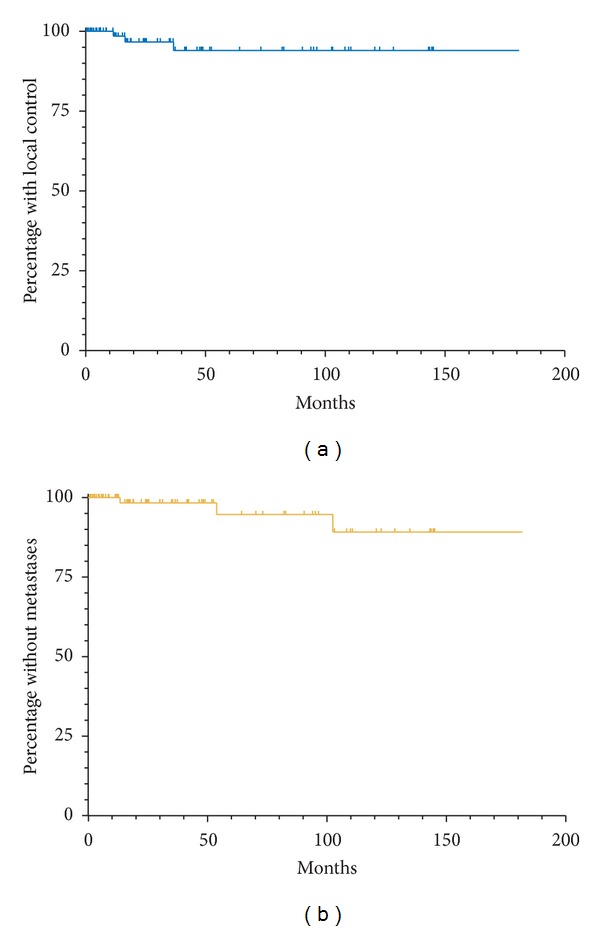
(a) Local control. (b) Freedom from metastatic disease.

Patient OS and FFP is displayed in Figure 1. Median follow-up for the 88 evaluable patients was 48.9 months (range 1–156 months). The five- and ten-year OS for our patient population was 90% and 79%, respectively (95% C.I. 79–95%, and 64–88%, resp.). The five- and ten-year FFP was 88% and 83%, respectively (95% C.I. 73–95%, and 62–93%, resp.). The local control and metastasis free rates are displayed in Figure 2. Five- and ten-year local control rates were 94% at both time points (95% C.I. 82–98% and 82–98%, resp.), and metastasis free rates were 95 and 89% at five and ten years (95% C.I. 79–99% and 67–97%, resp.). Local failure was seen in only three patients, one of whom underwent enucleation, and two patients underwent transpupillary thermotherapy. Fifteen patients also underwent laser photocoagulation at the time of plaque implant.

A univariate analysis was then performed for factors affecting FFP. Factors evaluated included patient age, sex, dose to the tumor apex, and tumor characteristics including COMS classification, AJCC stage, tumor location by equatorial position, involvement of the ciliary body, tumor height, and plaque margin on the tumor. Dose was dichotomized both to doses to the apex of < or ≥85 Gy as well as < or ≥90 Gy. Results are displayed in Table 3. There were no statistically significant variables impacting FFP seen. With no significant variables on univariate analysis, a multivariate analysis was not performed.

Overall, treatment was well tolerated. Cataract formation and radiation retinopathy were seen in 25% and 22% of patients, respectively. Glaucoma was noted after treatment in 6% of patients. Optic neuropathy was seen in 3% of patients. Other complications noted included mild/transient diplopia, retinal detachment/hemorrhage, and conjunctival irritation. Decreased visual acuity was the most common functional change seen, with 64% of patients maintaining best corrected vision of 20/200 or better. Results are displayed in Table 4.

The impact of dosimetric variables and select tumor characteristics on treatment side effects was then investigated. Variables were chosen in accordance to logical associations, and a blanket analysis of all known factors was not performed. A statistically significant correlation between lens dose and cataract formation was seen (*P* = 0.05). In addition, correlations between lens dose and glaucoma formation, as well as both tumor apex and 5 mm depth dose to radiation retinopathy, were also noted (*P* = 0.04, *P* = 0.01, and *P* = 0.04, resp.). No other dosimetric variables were statistically noted to have an impact on the incidence of side effects to treatment. Results are displayed in Table 5.

## 4. Discussion

The results of the COMS [3, 16] studies have well demonstrated that plaque brachytherapy is an acceptable treatment modality for medium sized tumors. The 5-year OS was 82% and 81%, and rates of distant metastases 91% and 89% for the patients receiving plaque brachytherapy and enucleation, respectively, with no statistical difference between treatment modality [16]. Longer term follow-up demonstrated that there continued to be no survival difference between these two treatment modalities [3], and in light of lower treatment morbidity plaque brachytherapy has become the predominant treatment modality [17].

While the COMS study has validated the implementation of plaque brachytherapy for medium sized tumors, the process is complex and special considerations must be undertaken in a multidisciplinary approach [6]. As such it is important to verify that the results obtained by the COMS study can be translated into clinical practice and that results are not limited to a few large institutions. Many of the largest studies to date have come from select institutions [7–10, 15, 18–21], and reports from other clinical practices are much more limited.

The results of our study also demonstrate excellent outcomes for the utilization of plaque brachytherapy. Our 5- and 10-year OS rates were 90 and 79%, respectively. At the same time intervals, 88% and 83% remained free from either local failures or distant metastases. These results are similar to the COMS report [3], where 10-year OS for both groups cumulatively was 65%, and 10-year survival in the absence of metastatic disease was 83%, demonstrating that at least equivalent results can be obtained in a community practice based setting.

Our results demonstrated local control which plateaued after 3 years, with long term local control being 94%. These results compare favorable to the COMS study, where 5-year local control rates were reported as 89.7% [22]. Several institutions have shown similar control rates, ranging from 81 to 93% [12, 15, 21, 23], and, in a study by Leonard et al. [11], a literature review was performed where the 5-year local control average was thought to be 88.1% for I-125 plaques. However, it was noted in the same study that long term local control has not been as well established, with rates confined almost entirely to select few studies [7, 18, 19, 24]. While a limited number of patients were evaluable for long term follow-up in our study (for LC only 11 patients at 10 years), the 10-year local control rate was 94%, with only 3 patients having experienced a true local failure. In addition, these failures all occurred within the first 3 years of therapy. Nevertheless, other studies have continued to show late recurrences after 5 years [18], and our study also demonstrated an even distribution of metastases over the time period evaluated, indicating the need for continued follow-up and close monitoring long term.

In our study, no significant prognostic factors were seen to impact FFP rates. This includes patient demographic variables, tumor characteristics, plaque margin, and tumor apex dose. In contrast, in a report by Leonard et al. [11], multiple factors including gender and apical height were predictive of local failure on univariate analysis. Similarly, in a report by Jensen et al. [12], tumor diameter, tumor height, and tumor apex dose, as well as other factors, were predictive of either local failure or distant metastasis. In the COMS study [3], tumor diameter, apical height, and equatorial position were all predictive of development of distant metastasis. While these results certainly contrast with our study, the lack of prognostic impact can be explained fairly readily by the low instances of either local failures or distant metastases, and these numbers were likely not adequate to demonstrate any statistical significance regarding these outcomes in the absence of higher patient numbers.

The availability of studies evaluating a dosimetric impact on treatment toxicity is relatively limited. Jensen et al. [12] performed evaluations regarding both total doses and dose rates on both treatment outcomes and toxicity. They found associations in visual acuity or blindness with total doses and dose rates to the macula, optic disk, and lens. Gündüz et al. [8] on univariate analysis found maculopathy to be associated with apex dose rate, and papillopathy to be associated with apex dose rate and optic disk dose. Jones et al. [13] noted association between dose rates to the macula and visual decline after treatment. Stack et al. [14] noted relationships between dose and development of maculopathy and cataract formation.

The results of our study also demonstrated causal relationships between total dose to ocular structures and treatment toxicity. A statistical increase in cataract formation was noted with increased mean lens dose (*P* = 0.05). This is not surprising, as the association between lens dose and cataract formation is well known [25, 26]. In addition, a relationship between lens dose and glaucoma formation was also noted (*P* = 0.04). While the rates of radiation induced glaucoma in our study were relatively low, 6%, neovascularization was noted in almost 50% of the cases, and a causal relationship of neovascularization caused by radiation induced ischemia, and subsequent neovascular glaucoma has been noted [27]. Lastly, an association between both 5 mm depth dose and tumor apex dose to retinopathy was also observed (*P* = 0.01 and *P* = 0.04, resp.), indicating a correlation between increased prescription dose and the development of retinopathy. Overall, these results are consistent with institutional reports of the dose dependence of treatment complications, and while the exact relationships vary to some degree between our study and other published reports, it does seem to indicate that causal relationships do exist.

At the same time, while dose-dependent toxicities may be established, this should not necessarily change clinical practice. Tumor control is still the primary objective, and a reduced tumor dose or dose rate to improve toxicity might result in poor control. While the results of our study do not demonstrate any impact on FFP with dose thresholds of 85 Gy or 90 Gy, other dose effects have been noted. In the aforementioned study by Jensen et al. [12], decreased total dose to the tumor apex of <100 Gy and dose rates of <90 cGy/hr, while both associated with improved toxicity, were also associated with increased rates of metastases, and other studies have also demonstrated similar associations [8, 15]. While the actual dose and rate thresholds vary in the literature, a threshold does seem to exist. As a result, while dose-dependent toxicity relationships are present, clinical benefits are likely in the realm of improved patient counseling rather than tailored treatment.

Limitations to our study certainly exist. Not all patients had follow-up data available, especially with longer follow-up approaching 10 years, as indicated by the lower number of patients for whom primary outcomes and treatment complications were evaluable. In addition, with low rates of recurrences and metastases, many more patients would be required to fully establish relationships between dosimetric and tumor characteristics and treatment outcomes. This same critique applies to evaluating causal factors for treatment complications. In addition, it is a retrospective study, and limitations such as loss to follow-up, completeness of treatment records, record uniformity, and variations in follow-up monitoring all apply. Nevertheless, it still serves as a valuable marker of clinical practice in the modern era.

In conclusion, this series continues to demonstrate excellent and reproducible clinical outcomes for plaque brachytherapy for uveal melanomas, with satisfactory long term control. Local recurrences are quite rare, and treatment is well tolerated with decreases in visual acuity being the most common effect of treatment. Relationships appear to exist between treatment toxicity and doses to intraocular structures. Further evaluation would be warranted to fully evaluate these relationships.

## Figures and Tables

**Table 1:** Patient and tumor characteristics.

	All treated patients	Patients with available F/U
Number of Patients	107	88
Age at diagnosis (yr)		
Median	65	65
Range	22–93	22–93
Sex		
Male	60 (56%)	48 (55%)
Female	47 (44%)	40 (45%)
Laterality		
Left	56 (52%)	47 (53%)
Right	51 (48%)	41 (47%)
Preexisting Conditions		
Hypertension	50 (47%)	40 (45%)
CVS Disease	16 (15%)	12 (14%)
Diabetes	15 (14%)	10 (11%)
Smoking History	17 (16%)	18 (20%)
COMS Classification		
Small	13 (12%)	12 (14%)
Medium	91 (85%)	74 (84%)
Large	3 (3%)	2 (2%)
Stage		
T1a	39 (36%)	32 (36%)
T1b	1 (1%)	1 (1%)
T2a	43 (40%)	38 (43%)
T2b	5 (5%)	5 (6%)
T3a	15 (14%)	9 (10%)
T3b	3 (3%)	3 (3%)
T4a	1 (1%)	0 (0%)
T4b	0 (0%)	0 (0%)
Equatorial position		
Anterior	8 (7%)	7 (8%)
Posterior	70 (65%)	58 (66%)
Spanning	25 (23%)	20 (23%)
Unknown	4 (4%)	3 (3%)

F/U: follow-up; COMS: collaborative ocular melanoma study; CVS: cardiovascular.

**Table 2:** Delivered dosimetric parameters.

	Median dose (Gy)	Range (Gy)
Tumor apex	85.90	83.72–147.20
Inner sclera	248.95	99.99–1132.30
Opposite retina	6.44	2.84–23.30
Macula	46.51	8.17–174.47
Optic disc	39.78	8.18–238.70
Lens center	13.31	4.53–112.10

Gy: gray.

**Table 3:** Univariate analysis on factors impacting.

	HR	95% C.I.
Age		
<60	Referent	
≥60	2.56	0.58–11.26
Sex		
Male	Referent	
Female	2.00	0.44–9.06
COMS classification		
Small	0.84	0.11–6.13
Medium/large	Referent	
T-stage		
1	0.68	0.13–3.69
2	Referent	
3	1.11	0.16–7.75
4	2.71	0.16–7.73
Equatorial position		
Anterior	1.07	0.21–5.46
Spanning	1.66	0.11–25.64
Posterior	Referent	
Ciliary body involvement		
Not involved	Referent	
Involved	1.89	0.12–28.75
Tumor height		
<5 mm	Referent	
≥5 mm	1.04	0.23–4.67
Plaque margin		
<3.5 mm	2.79	26.08–0.30
≥3.5 mm	Referent	
Apex dose		
<85 Gy	*0.33 *	0.03–3.32
≥85 Gy	Referent	
<90 Gy	Referent	
≥90 Gy	2.13	0.46–9.76

FFP: freedom from progression; COMS: collaborative ocular melanoma study.

**Table 4:** Side effects from treatment.

Side effects	
Pts with evaluable f/u	88
Clinically useful vision	56 (64%)
Cataracts	21 (24%)
Radiation retinopathy	19 (22%)
Optic neuropathy	3 (3%)
Glaucoma	5 (6%)

**Table 5:** Analysis of factors predicting for treatment side effects.

	Absent (mean)	Present (mean)	*P* value
Cataracts			
Lens dose (Gy)	14.67	24.64	*0.05 *
Glaucoma			
Lens dose (Gy)	17.12	32.11	*0.04 *
Retinopathy			
5 mm depth dose (Gy)	78.48	108.6	*0.01 *
Inner dclera dose (Gy)	280.65	333	*0.14 *
Tumor size (mm)	10.47	10.52	*0.93 *
Tumor height (mm)	4.26	4.68	*0.40 *
Dose apex (Gy)	91.19	96.99	*0.04 *
Optic neuropathy			
Macula dose	62.51	95.7	*0.22 *
Optic disk dose	47.06	48.83	*0.93 *
Clinically useful vision			
Tumor size (mm)	11.22	10.28	*0.10 *
5 mm depth dose (Gy)	98.34	83.48	*0.15 *
Optic disk dose (Gy)	55.25	44.81	*0.25 *
Inner sclera dose (Gy)	343.67	280.75	*0.10 *
Macula dose (Gy)	75.49	60.31	0.20
